# Scientific writing and publishing: its importance to radiologists

**DOI:** 10.2349/biij.3.3.e55

**Published:** 2007-07-01

**Authors:** WCG Peh

**Affiliations:** Department of Diagnostic Radiology, Alexandra Hospital, Singapore

**Keywords:** Academic radiology, manuscript preparation, medical publication, professional development, research, scientific writing

## Abstract

Scientific writing and publication marks the endpoint of research that has been performed, completed, peer reviewed and accepted, and complements teaching and training, clinical service and patient care. Writing has numerous benefits, one of the most important ones being the inherent training undertaken to better appreciate and evaluate the published work of others. Effective scientific writing is an important component of a radiologist’s practice, and should be cultivated at an early stage of the career.

## INTRODUCTION

The reasons for scientific writing range from noble to base reasons. Topping the list is altruism, where one writes for the pleasure derived from the creative activity of writing and from sharing one’s intellectual pursuits, as well as for the desire to advance human knowledge for the benefit of mankind. For these authors, writing is a channel for expressing the joy of scientific discovery. At the bottom of the list, writing may be considered by some to be a chore where getting published is a ‘necessary evil’ in order to fulfil certain specific minimum requirements, e.g. for getting a job or a promotion.

Writing is one of the marks of human civilisation - an advanced means by which humans communicate with each other. A published article is indisputable evidence of research that has been performed, completed, and accepted by peers. Publication is also an indicator of achievement of a certain academic standard. Besides communication of a finalised piece of research, the written work is the basis for further opinions, views and critiques from fellow professionals and academics separated by time and distance. Most importantly, it represents the only permanent record of scientific work that has been completed.

## TYPES OF SCIENTIFIC WRITING

Scientific writing encompasses a whole range of forms, including theses, books, book chapters, grant applications, course syllabi, proffered abstracts, and journal articles. For the medical community, publication in peer-reviewed scientific journals that are indexed in a major database such as PubMed (a service of the US National Library of Medicine that includes over 17 million citations from MEDLINE and other life science journals for biomedical articles) carries the highest weight, as accepted manuscripts are peer-reviewed and widely accessible internationally.

## BENEFITS OF WRITING

For doctors, and specifically radiologists, the benefits of scientific writing can be grouped into the following headings:

- Career
- Professional
- Institutional
- Practical
- Radiologist-specific

### Career benefits

The most compelling reason for many doctors to start writing is to fulfil specific job requirements by employers (e.g. hospitals or universities). These include initial appointment to an academic position, renewal or confirmation of that appointment, promotion to a higher level appointment, and granting of tenure. In some public hospitals in Singapore, having publications in a recognised journal is a requirement for appointment as a consultant. Other career benefits include professional accreditation, continuing medical education (CME) accreditation, and application for membership in prestigious learned societies. In Singapore, where obtaining a minimum number of CME points over a two-year cycle is compulsory for renewal of a doctor’s practising certificate, the Singapore Medical Council awards CME points for successful publication.

### Professional benefits

Publications can be regarded as an international currency that transcends political borders. For young doctors, having published articles in internationally reputable journals are a great help when applying for positions in foreign institutions and for overseas fellowships. For more established doctors, publications enable them to gain recognition and acknowledgement as experts in a particular field at national and international levels. Invitations to lecture at scientific meetings and refresher courses, and appointments as consultants to external agencies, expert panels and advisory boards, and to reviewer and editorial boards, are among the benefits of this enhanced professional reputation.

Having topic-focused publications is also regarded as attainment of a certain standard of scholarly endeavour by several prestigious invitation-only international learned societies. From the academic point of view, writing and getting published improves one’s prospects of being successful in applications for research funding, extension of funding, and to obtain further funding. Grant-awarding bodies usually closely examine the publication track record of the applying investigators, when considering dispensation of funds.

The discipline imposed by scientific study, research and writing increases the depth of knowledge in the subject being investigated. This knowledge sharpens clinical skills and facilitates teaching of students and postgraduates. Through scientific writing and publication, the author achieves expertise and eventually becomes acknowledged as an authority by academic peers in similar fields of endeavour.

### Institutional benefits

Publication in peer-reviewed journals is arguably the most important means to get international recognition for an individual, department, hospital, and university. The author’s country, and even the region, may also derive benefit from published work, particularly if it is on a topic of major importance. Besides educating peers locally and regionally, publications on subjects of relevance or common interest also serve as a conduit to establish links with other centres, with potential for clinical referral, training and research opportunities.

Many government bodies and academic institutions use publications as a measure of academic productivity. Published papers not only contribute to an institution’s academic prestige and standing; for individual academic cost centres and departments, they may be linked to, and have a critical influence on, the annual budget allocation.

### Practical benefits

The most important practical reason for knowing how to write is probably the benefit derived from the inherent training to be discriminatory and critical during the process of manuscript preparation. Scientific writing entails the discipline of performing a complete literature search, gathering and analysing data, and writing and revising numerous versions of a manuscript. Following the satisfaction of having their own manuscripts accepted, authors will be better positioned to appreciate what is written in journals and other scientific publications. If asked to act as a journal manuscript reviewer, it is strongly recommended that all doctors should accept the invitation to undertake this valuable learning process.

With the huge amount of information now available in so many journals and other print material, it is vital for all professionals and academics to be able to judge the quality and reliability of published work. If one has published and appreciates the writing, reviewing and editing process, then one will be better able to read articles with the correct scientific and critical technique, and assess them for their true worth. Being able to provide a critical evaluation and learned judgement of what is written are skills that will produce a better clinical doctor. After all, medical practice is a knowledge-based profession. Patients always want to be seen by the most knowledgeable and up-to-date doctor.

### Radiologist-specific benefits

Diagnostic radiology is rapidly evolving. To be able to provide the best imaging service to patients, radiologists have to be constantly up-to-date and able to influence clinicians. Most clinicians do not have in-depth knowledge of, or formal training in, imaging and interventional radiology techniques, but they may be compelled to move into the radiologist’s turf, if radiologists are not providing the service to their requirements and satisfaction. The reality of radiology clinical practice is that effective communication is required. Unlike most of our clinical colleagues, our ’clients’ are not laypersons but highly-qualified doctors, usually specialists and sub-specialists. Radiologists are often referred to as the ’doctor’s doctors’, and must therefore strive to live up to this moniker.

For radiologists, scientific writing is important in different phases of a radiological career. In the initial four-to-six year training period, the importance of written communication is recognised by its incorporation into the examination and accreditation system. Most radiology examinations include a written component where the candidate has to write quickly and succinctly within a short period of time. This usually takes the form of a film reporting session. Many professional Masters in Radiology courses have a mini-thesis component, incorporating all the elements of basic research techniques and manuscript preparation. Many awarding bodies have a requirement for publication(s) as part of the exit assessment or examination.

Obtaining the exit radiological qualification and passing the various examinations may be considered a licence to continue the life-long learning process unsupervised. Written communication is an integral part of daily radiology practice. Radiologists are judged by their reports, in terms of style, accuracy and completeness. The reports should be of practical usefulness, contributing to the diagnosis and management of patients. As the radiology reports may also be read by peers from other institutions, including foreign ones, they should be clear, concise and written in a universally-understood format. Being proficient in scientific writing is therefore a necessity if one aims to be a competent international-standard radiologist.

## SUMMARY

Writing is the most important means for communicating scientific work. Research and publication complement teaching and training, clinical service and patient care. There are many reasons for writing, one of the most important of which is the inherent training undertaken to better appreciate and evaluate the published work of others. Effective scientific writing is an important component of a radiologist’s practice. Trainees should be encouraged to start early, and senior members of our profession should act as role models and provide support.

